# Combined Impact of Excess Zinc and Cadmium on Elemental Uptake, Leaf Anatomy and Pigments, Antioxidant Capacity, and Function of Photosynthetic Apparatus in Clary Sage (*Salvia sclarea* L.)

**DOI:** 10.3390/plants11182407

**Published:** 2022-09-15

**Authors:** Anelia Dobrikova, Emilia Apostolova, Ioannis-Dimosthenis S. Adamakis, Anetta Hanć, Ilektra Sperdouli, Michael Moustakas

**Affiliations:** 1 Institute of Biophysics and Biomedical Engineering, Bulgarian Academy of Sciences, 1113 Sofia, Bulgaria; 2 Section of Botany, Department of Biology, National and Kapodistrian University of Athens, 15784 Athens, Greece; 3 Department of Trace Analysis, Faculty of Chemistry, Adam Mickiewicz University, 61-614 Poznan, Poland; 4 Institute of Plant Breeding and Genetic Resources, Hellenic Agricultural Organisation–Demeter, Thermi, 57001 Thessaloniki, Greece; 5 Department of Botany, Aristotle University of Thessaloniki, 54124 Thessaloniki, Greece

**Keywords:** leaf morphology, essential elements, chlorophyll, total phenolic content, anthocyanins, photosystems, oxygen-evolving complex, phytoextraction, heavy metal toxicity

## Abstract

Clary sage (*Salvia sclarea* L.) is a medicinal plant that has the potential to be used for phytoextraction of zinc (Zn) and cadmium (Cd) from contaminated soils by accumulating these metals in its tissues. Additionally, it has been found to be more tolerant to excess Zn than to Cd stress alone; however, the interactive effects of the combined treatment with Zn and Cd on this medicinal herb, and the protective strategies of Zn to alleviate Cd toxicity have not yet been established in detail. In this study, clary sage plants grown hydroponically were simultaneously exposed to Zn (900 µM) and Cd (100 μM) for 8 days to obtain more detailed information about the plant responses and the role of excess Zn in mitigating Cd toxicity symptoms. The leaf anatomy, photosynthetic pigments, total phenolic and anthocyanin contents, antioxidant capacity (by DPPH and FRAP analyses), and the uptake and distribution of essential elements were investigated. The results showed that co-exposure to Zn and Cd leads to an increased leaf content of Fe and Mg compared to the control, and to increased leaf Ca, Mn, and Cu contents compared to plants treated with Cd only. This is most likely involved in the defense mechanisms of excess Zn against Cd toxicity to protect the chlorophyll content and the functions of both photosystems and the oxygen-evolving complex. The data also revealed that the leaves of clary sage plants subjected to the combined treatment have an increased antioxidant capacity attributed to the higher content of polyphenolic compounds. Furthermore, light microscopy indicated more alterations in the leaf morphology after Cd-only treatment than after the combined treatment. The present study shows that excess Zn could mitigate Cd toxicity in clary sage plants.

## 1. Introduction

Contamination and accumulation of heavy metals in the environment, where their bioavailability may be high, is an important environmental problem globally [1,2]. Some metals such as cadmium (Cd) have no metabolic role in plants and can induce toxicity symptoms depending on the concentrations, plant species, etc. [3,4]. The presence of Cd at high concentrations in plants can reduce plant growth due to decreased chlorophyll content and photosynthetic rate [5,6,7,8]. Cd toxicity can also cause changes in the lipid composition of thylakoid membranes and in the chloroplast ultrastructure. Moreover, it can cause anatomy disorders, chlorosis or necrosis, and can even cause plant death [2,8,9,10,11]. Additionally, Cd-induced toxicity and cell damage are caused by the accumulation of reactive oxygen species (ROS) [12,13,14]. Therefore, it is important to study the regulatory mechanisms and the plant responses to Cd toxicity, which is essential to minimize risk in the food chain and further facilitate phytoremediation practices in Cd-contaminated soils. 

Other elements, such as zinc (Zn), copper (Cu), iron (Fe), manganese (Mn), calcium (Ca), and magnesium (Mg), are considered as essential trace elements or mineral nutrients for many structural and biochemical functions, such as plant growth, electron transport, oxidation-reduction reactions, and many other metabolic activities in plants [15]. In particular, Zn is an important element, since it regulates physiological and metabolic processes, i.e., it is a cofactor of many enzymes involved in respiration and photosynthesis, in biosynthesis of plant growth hormones, etc. [16,17,18,19]. Moreover, at low concentrations (below 300 µg g^−1^ DW), Zn can favorably influence the growth, development, and the performance of both photosystems in plants [14,20]. However, at high concentrations, it can compete with other metals at absorption sites, reducing leaf area, causing phytotoxicity and oxidative stress, chlorosis, and even plant necrosis [21,22]. In addition, Zn is regarded as the principal micronutrient to ameliorate the toxic effect of Cd in plants and to limit its entry into the food chain [23]. The addition of Zn to Cd-containing soils or nutrient solutions has been proposed to be successful in reducing Cd accumulation in crop plants [24,25], but plant responses vary with genotype and the dose and duration of the Zn and Cd exposure [14,22,26]. 

The accumulation of heavy metals in some medicinal plants, collected near industrial, mining, and farming sites, was found to be at high levels [15]. Furthermore, the aromatic plants have recently attracted much attention as an excellent option for reclamation and sustainable phytoremediation due to their increased resistance and the low threat of toxicity of the final products, as there is no risk of hazardous metal accumulation during the extraction of essential oils from these plants [27]. The medicinal and aromatic plant clary sage (*Salvia sclarea* L.) is intensively exploited due to its broad medicinal properties. It is native to many Mediterranean countries and is cultivated as a source of essential oils for applications in human medicine or perfumery products [28,29,30]. Our previous investigations suggested that clary sage might be introduced as a Cd-hyperaccumulator plant when exposed to high concentrations of Cd (100 µM) for 8 days in a nutrient solution but undergoes damage of thylakoid membrane organization and consequent impairment of the photosynthetic electron-transport chain [8,31]. Treatment with excess (900 µM) Zn revealed that clary sage tolerates high toxic levels of Zn in its tissues and retains photosynthetic function, it even stimulates the activities of photosystem I (PSI) and photosystem II (PSII) [32]. Another recent study on co-exposure of clary sage to high Cd and Zn concentrations showed that the application of excess Zn effectively alleviated Cd-induced toxicity by reducing Cd uptake by half and reducing its concentration in the leaves [33]. Concomitantly, it has also been demonstrated that excess Zn mitigates Cd-induced oxidative stress, partially retains the chloroplast ultrastructure of clary sage, and protects the PSII photochemistry as it reduces the excess excitation energy at PSII (EXC). Clairvil et al. [14] also showed that in vitro co-exposure to Cd and Zn in *Alternanthera tenella* plants can enable Cd-induced detoxification and the proper functioning of the photosynthetic apparatus. 

The recently proposed potential of clary sage (*S. sclarea*) for the phytoextraction of Cd and Zn from heavy metal-polluted soils [31,32] demonstrates the need to elucidate the molecular mechanisms and tolerance strategies of this medicinal herb when simultaneously exposed to high concentrations of Cd and Zn, as industrial and agricultural soils are generally contaminated by several heavy metals. Thus, the objective of the current study was to provide a detailed exploration of nutrient elemental uptake, the degree of membrane damage and membrane fluidity, the leaf anatomy, the antioxidant activity, the total phenolic and pigment contents, and the functions of the two photosystems and the oxygen-evolving complex (OEC) in clary sage under co-exposure to excess Zn and Cd under hydroponic conditions. These investigations provide additional knowledge concerning the defense mechanisms and the role of excess Zn in reducing Cd toxicity in this medicinal plant. Keeping in mind that the quality of essential oils is not affected by heavy metals [34], the research in this study is important to ensure the protection and sustainable cultivation of this valuable dual-use plant for pharmaceutical applications and the phytoremediation of soils contaminated with heavy metals.

## 2. Materials and Methods

### 2.1. Plant Material, Growth Conditions, and Heavy Metal Treatments

Seeds of clary sage (*Salvia sclarea* L.) kindly provided by Bio Cultures Ltd. (Karlovo, Bulgaria) were used for the experiments. After germination on soil for about a month, the seedlings were transferred to continuously aerated 1 L containers and changed every 3 days with a modified half strength (½) Hoagland nutrient solution containing the following: 1.5 mM KNO_3_, 1.5 mM Ca(NO_3_)_2_, 0.5 mM NH_4_NO_3_, 0.5 mM MgSO_4_, 0.25 mM KH_2_PO_4_, 50 μM NaFe(III)EDTA, 23 μM H_3_BO_3_, 5 μM ZnSO_4_, 4.5 μM MnCl_2_, 0.2 μM Na_2_MoO_4_ and 0.2 μM CuSO_4_ (pH 6.0, EC = 1.03 dS m^−1^) as described before [31]. The growth room conditions were 14/10 h day/night photoperiod and 24/20 °C day/night temperature, with 200 ± 20 µmol photons m^−2^ s^−1^ photon flux density. After a month on hydroponic culture, containers with plants were divided into three groups, and each group was subjected for 8 days either with: (1) ½ Hoagland nutrient solution (control); (2) ½ Hoagland nutrient solution with 100 μM Cd (supplied as CdSO_4_) plus 900 μM Zn (supplied as ZnSO_4_) (combined Cd + Zn treatment); or (3) ½ Hoagland nutrient solution with 100 μM Cd (Cd-only treatment). The concentrations and duration of Cd and Zn treatments were based on our previous studies [31,32]. When *S. sclarea* plants were exposed to 100 μM Cd for 8 days, an inhibition of PSII functionality was noticed [31], but an enhanced PSII functionality occurred for the same period of exposure to 900 μM Zn [32]. Thus, in this study, we used the simultaneous exposure to 100 μM Cd and 900 μM Zn for 8 days to observe any alleviative effect of Zn against Cd toxicity.

### 2.2. Determination of Trace Element Concentrations by Inductively Coupled Plasma Mass Spectrometry (ICP-MS)

The trace element determination in clary sage tissues after the 8-day treatments (control, Cd only, and Cd + Zn) was performed as described previously [31]. Dry samples were digested in a closed-vessel microwave system (Ethos One, Milestone Srl, Sorisole, Italy). Digested tissue samples were analyzed using inductively coupled plasma mass spectrometry (ICP-MS) model ELAN DRC II (PerkinElmer Sciex, Toronto, Canada), and the conditions, instrumental settings, calibration solutions, data validation, and validation parameters were as described in [31]. Elemental analysis was performed for Fe, Ca, Mg, Cu, and Mn ions. 

Translocation factor (TF) was calculated as the ratio of the elemental concentration in shoots to the concentration in roots [32].

### 2.3. Light Microscopy and Leaf Thickness

Leaf segments from variously treated plants were chemically fixed, sectioned and stained, and the sections were observed as reported in Dobrikova et al. [31]. Leaf thickness measurements from cross leaf sections, obtained from five individual leaves in each treatment, were made using the ZEN 2.0 software (Carl Zeiss, Munich, Germany) according to the manufacturers’ instructions. 

### 2.4. Determination of Membrane Stability Index, Lipid Peroxidation, and Total Antioxidant Capacity

The leaf cell membrane stability index (MSI) was estimated from the electrolyte leakage measurements described previously [35]. Fully expanded leaves from different plants were cut into pieces (about 3–4 cm^2^) and then incubated in tubes with 40 mL distilled water for 24 h on a shaker at room temperature. After that, the electrical conductivity of the solutions (EC1) was measured using a conductometer (Hydromat LM302, Witten, Germany). Thereafter, the samples were boiled for 30 min and cooled to room temperature to determine the final electrical conductivity (EC2). The MSI values were calculated using the following equation: MSI = [1 − (EC1/EC2)] × 100.

The determination of the levels of lipid peroxidation (LP) was made by estimating the MDA content as described by Mostofa et al. [36]. The total antioxidant capacity was evaluated by both DPPH• (free radicals scavenging activity) and FRAP (ferric-reducing antioxidant power) assays of leaf methanol extracts from differently treated clary sage plants as described in [37]. DPPH radical is a stable radical with a maximum absorption at 517 nm that can readily undergo reduction by an antioxidant and the degree of de-coloration indicates the scavenging potentials of the antioxidants. The results were expressed as the inhibition of free DPPH radical in percentages using the following equation: I (%) = (A_blank_ − A_sample_/A_blank_) × 100, where A_blank_ is the absorbance of the control reaction (containing all reagents except the plant extracts), and A_sample_ is the absorbance of the test extract. The FRAP method is based on the reduction of a ferric-tripyridyl triazine complex to its ferrous-colored form in the presence of antioxidants. For FRAP analysis samples were measured at 595 nm and the antioxidant potential of extracts was determined from a standard curve, plotted using the FeSO_4_ linear regression, and values were expressed as μmol Fe^2+^ per g of dried weight (DW). All assays were determined in triplicate.

### 2.5. Analysis of Leaf Pigments and Total Phenolic Content

The measurements of total chlorophyll (Chl *a+b*) and carotenoids (Car) were performed as described previously in detail [31] using a spectrophotometer (Specord 210 Plus, Ed. 2010, Analytik Jena AG, Germany). For an estimation of the anthocyanin and total phenolic contents, the frozen leaf samples (0.1 g) were extracted with 10 mL acidified methanol (1% HCl) in darkness at 0–4 °C for 2 days. The total phenolic content was determined using the Folin–Ciocalteu’s colorimetric method and measured spectrophotometrically at 765 nm according to the method of Sripakdee et al. [38]. The phenolic content was expressed as mg of gallic acid equivalent (GAE) per g fresh weight (FW) of leaf tissues. Anthocyanins were estimated spectrophotometrically at 535 and 657 nm as described in Dobrikova et al. [31]. Anthocyanin content was expressed as mg of cyanidin-3-glucoside equivalent per g FW. The mean values (±SD) were averaged from three independent treatments with three repetitions for each treatment.

### 2.6. Chlorophyll Fluorescence Analysis

Dark-adapted (20 min) leaves from control, 100 μM Cd-, and 100 μM Cd + 900 μM Zn-treated *S. sclarea* plants were used for the chlorophyll fluorescence analysis using an Imaging PAM M-Series system (Heinz Walz Instruments, Effeltrich, Germany) as described in detail previously [39]. The chlorophyll fluorescence parameters calculated by the Imaging Win V2.41a software (Heinz Walz GmbH, Effeltrich, Germany) were the maximum efficiency of PSII photochemistry (*Fv*/*Fm*), the relative PSII electron transport rate (ETR = ΦPSII × PAR × c × abs, where PAR is the photosynthetically active radiation, c is 0.5, and abs is the total light absorption of the leaf taken as 0.84) [40], and the efficiency of the oxygen-evolving complex (OEC) on the donor side of PSII (*Fv/Fo*) [41,42].

### 2.7. Oxygen Evolution Measurements

Flash-induced oxygen evolution of isolated thylakoid membranes from clary sage leaves after 8-day treatment of plants (control, Cd-only, and combined (Cd + Zn) treatment) was measured using a home-built Joliot-type polarographic oxygen rate electrode without an artificial electron acceptor [43]. The thylakoid membranes were isolated and the flash-induced oxygen yields (*Y_i_*) were measured as described in [7]. According to the Kok’s model, the Mn_4_Ca cluster of OEC passes through five intermediate redox states (S_0_–S_4_) in the same PSII reaction center to produce one oxygen molecule [44]. In the darkness, only the S_0_ and S_1_ states are stable, so the initial S_0_/S_1_ state distribution and the percentage of misses (α) were calculated from the experimentally obtained flash-induced oxygen yields using a computer program of the Kok’s model [7,31]. The parameter S_B_ (corresponding to the amount of the blocked PSII centers) was obtained using extended kinetic version of the Kok’s model (for details see [45,46]). The S_B_ could go back to the S_0_ state with liberation of the accepted oxygen molecules: K_A_.S_B_ → S_0_ + O_2_, where K_A_ is the activation rate constant [45,46].

### 2.8. Measurements of P700 Redox State and Microviscosity

The oxidation of the PSI reaction centers (P700) to P700^+^ and a consequent dark reduction of P700^+^, an indicator of redox changes of P700, were measured in vivo by following the far-red (FR) light-induced absorbance transients at 830 nm (*ΔA*_830_) using a PAM-101/103 fluorometer (Walz, Effeltrich, Germany) equipped with an ED-800T emitter-detector. The measurements were performed on dark-adapted leaves using FR light supplied by a photodiode (102-FR, Walz). The extent of the FR-induced oxidation of P700 to P700^+^ was estimated as the relative ratio *ΔA/A_830_*, where Δ*A* was the FR-induced absorbance change and *A_830_* was the absorbance in the dark. The capacity of the cyclic electron flow around PSI was estimated by the kinetics of the P700^+^ dark reduction after switching off the FR light, as shown previously [7,47].

The relative microviscosity (*η*) of thylakoid membranes was investigated by means of fluorescence polarization of the fluorescent lipophilic probe 1,6-diphenyl-1,3,5-hexatriene (DPH), using a fluorescence spectrometer (JASCO FP-8300, Tokyo, Japan) as described in the work of Dobrikova and Apostolova [48].

### 2.9. Statistics

The mean values (±SD) were calculated from three independent treatments with three replicates each and the statistically significant differences among the means were determined using a one-way ANOVA analysis. Mean values were considered statistically different after Fisher’s least significant difference post-hoc test by using the Origin 9.0 software (OriginLab, Northampton, MA, USA). Prior performing ANOVA, the assumptions for normality of the data distribution (by Shapiro–Wilk’s tests) and homogeneity of variances (by Levene’s test) were checked as described previously [31]. The statistical analysis for each measurement is reported in the corresponding figure and table legend.

## 3. Results

### 3.1. Trace Elemental Content in Plant Tissues

**Figure 1:**
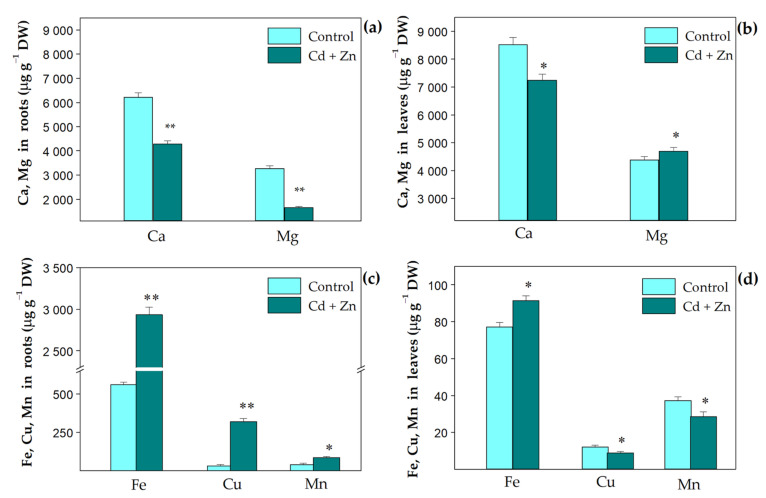
Contents of Ca, Mg (**a**,**b**), and Fe, Cu, Mn (**c**,**d**) in *Salvia sclarea* roots and leaves, respectively, after 8 days of exposure at 100 µM Cd with and without 900 µM Zn. Mean values (±SD, n = 3) were compared between control and treatment for the same mineral element using a one-way ANOVA, and the differences were considered statistically significant with * *p* < 0.05 and ** *p* < 0.01.

**Figure 2:**
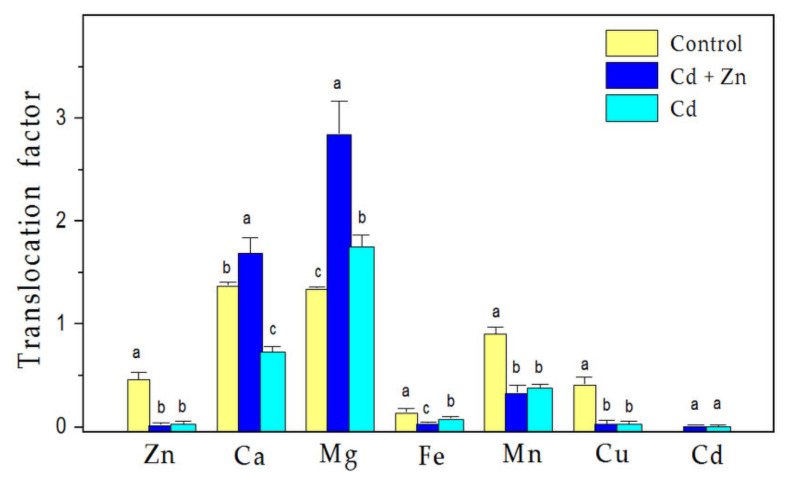
Changes in the translocation factors of the mineral elements in *Salvia sclarea* plants in response to 100 µM Cd treatment with and without excess (900 µM) Zn exposure for 8 days. Mean values (±SD, n = 3) were compared between the treatments for the same element performing one-way ANOVA analysis. Different letters indicate significant differences between the values for the same element at *p* < 0.05 using Fisher’s LSD post-hoc test.

The effects of the excess (900 µM) Zn supply to 100 µM Cd exposure on the uptake of selected nutrient elementals (Figure 1) and their translocation (Figure 2) in *S. sclarea* were studied. Compared to the control plants, the combined treatment with Zn and Cd resulted in a reduced accumulation of Ca ions in both roots and leaves (*p* < 0.01), while the accumulation of Mg decreased in the roots, but increased in the leaves (*p* < 0.05, Figure 1a,b). This was due to the more pronounced translocation of Mg ions to leaves (about two times) than that of Ca ions (about 1.2 times) (Figure 2). In addition, the translocations of Ca and Mg ions (also their leaf contents) were more significantly enhanced after the combined treatment than the control and Cd-only treatment (Figure 2). 

Additionally, the results demonstrated that the uptake of the other elements (Fe, Cu, Mn) was increased in the roots (Figure 1c), but their translocation to the leaves declined after both the combined and Cd-only treatments in comparison to the control (Figure 2). In the leaves, after the combined treatment, only an increased accumulation of Mg (by 8%, *p* < 0.05) and Fe (by 18%, *p* < 0.05) ions was observed in comparison with the control (Figure 1b,d). Data for the Cd-only treatment are given in Supplementary Table S1. 

### 3.2. Leaf Anatomical Features

**Figure 3:**
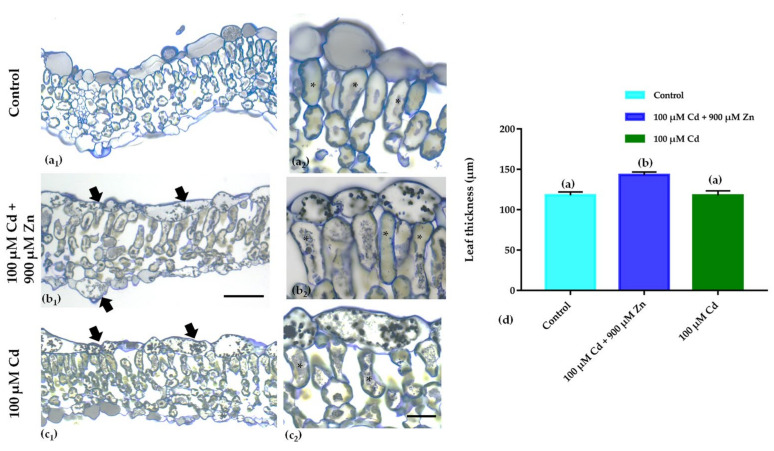
Leaf cross-sections (**a_1_**–**c_2_**) and leaf thickness measurements (**d**) of the control (**a_1_**,**a_2_**), 100 μM Cd + 900 μΜ Ζn (**b_1_**,**b_2_**), and 100 μΜ Cd (**c_1_**,**c_2_**) of 8-day-treated *Salvia sclarea* plants. Increased magnification images of the adaxial epidermis and palisade mesophyll cells are depicted in (**a_2_**,**b_2_**,**c_2_**). Upon 100μΜ Cd application, the cells of the adaxial epidermis were filled with osmiophilic granules (arrows in (**c_1_**,**c_2_**)), which in the combined (Cd + Ζn) treatment were also present in the abaxial epidermal cells (arrows in (**b_1)_**). Moreover, the palisade mesophyll cell structure was compromised in 100 μΜ Cd-treated plants (asterisks in (**c_2_**)), while after the combined exposure, it was similar to the control (asterisks in (**b_2_**) and (**a_2_**)). The combined treatment also seemed to increase leaf thickness (**d**). Scale bar in (**a_1_**,**b_1_**,**c_1_**): 50 μm. Scale bar in (**a_2_**,**b_2_**,**c_2_**): 10 μm. Different letters in (**d**) indicate significant differences (*p* < 0.05, n = 5).

The data showed that the Cd-only treatment affected *S. sclarea* leaf anatomy and a notable reduction in the intercellular spaces and an accumulation of osmiophilic granules to the adaxial epidermal cells was present (Figure 3c). The combined treatment (Cd + Zn) led to increased osmiophilic granule accumulation in both leaf epidermises (Figure 3b_2_). Moreover, Cd + Zn-treated leaves increased in thickness (Figure 3d), while they did not show any of the toxicity symptoms of Cd in the palisade mesophyll (compare Figure 3c_2_ with Figure 3b_2_), indicating that excess Zn application ameliorated Cd toxicity in *S. sclarea*. 

### 3.3. Membrane Damage and Antioxidant Capacity 

Measurements of the leaf cell membrane stability index (MSI) were performed to assess the extent of membrane damage in *S. sclarea* leaves in response to Cd exposure with or without excess Zn supply. The results showed that MSI was reduced by about 33% after the Cd-only treatment, while the combined treatment with excess Zn and Cd only caused a 15.6% decrease in comparison to the control (Table 1). The antioxidant capacity of leaf extracts from *S. sclarea* after the different treatments was evaluated by both the free radical scavenging activity (DPPH assay) and the total antioxidant activity (FRAP analysis), and the obtained results are presented in Table 1. The data revealed that the total antioxidant capacity (FRAP, reducing ability of antioxidants against oxidative stress) of *S. sclarea* leaf extracts increased after exposure to heavy metals in comparison to control conditions (Table 1). Moreover, among the extracts studied, after combined treatment with excess Zn and Cd, a higher antioxidant activity (157% of control values) was observed, followed by those treated with Cd alone (134% of control values). A similar trend was observed for the antioxidant activity assessed by DPPH. 

### 3.4. Photosynthetic Oxygen Evolution 

**Figure 4:**
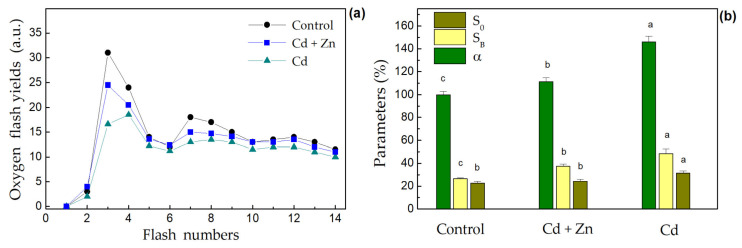
Effects of 100 µM Cd exposure for 8 days with and without excess (900 µM) Zn on: (**a**) oscillation patterns of the oxygen flash yields (*Y_i_*) of thylakoid membranes isolated from *S. sclarea* leaves and (**b**) S_0_—the amount of PSII centers in the most reduced state (S_0_ (%) = 100–S_1_), S_B_—the changes in the amount of blocked PSII centers presented as % of control (100% is 1.3 a.u.) and *α*–the misses (%). Different letters for the same parameter indicate significant differences between mean values (±SD, n = 3) assessed by one-way ANOVA analysis after Fisher’s LSD test (*p* < 0.05).

The oscillation patterns of oxygen yields induced by saturated flash sequences applied to dark-adapted thylakoid membranes isolated from the leaves of control, Cd-only, and combined (Cd + Zn) treated plants are presented in Figure 4a. 

It was observed that the inhibition of oxygen flash yields was less noticeable after the combined treatment than after the Cd-only treatment. From the detailed analysis of oscillation patterns performed using the extended kinetic version of the Kok’s model [45], values for the amounts of PSII centers in the initial dark-adapted S_0_ state (S_0_ (%) = 100–S_1_), the blocked PSII centers (S_B_) and the misses (*α*) were obtained (Figure 4b). The above-mentioned parameters are appropriate indicators for changes in the Mn cluster of OEC [7]. The results revealed that the Cd-only treatment caused a stronger increase in the number of PSII centers in the initial S_0_ state (by 22%), the blocked PSII centers (by 45%), and the misses (by 46%), while the combined exposure diminished these changes. Measurements of the PSII-dependent electron transport of the isolated thylakoid membranes with a Clark-type electrode showed a similar trend for effect of Zn supply on Cd-induced inhibition (data not shown). The analysis of oxygen flash yields revealed characteristic periodic sequences with a maximal oxygen evolution after the third flash (*Y_3_*). As compared to control plants, the oxygen yields slightly decreased after combined exposure and showed more pronounced damping after Cd-only exposure (Figure 4a). Moreover, the data demonstrated that the maximum observed after the 3rd flash (*Y_3_*) in the control and combined treatment plants shifted to the 4th flash after the Cd-only treatment (Figure 4a). 

### 3.5. Leaf Pigments and Total Phenolic Content

**Figure 5:**
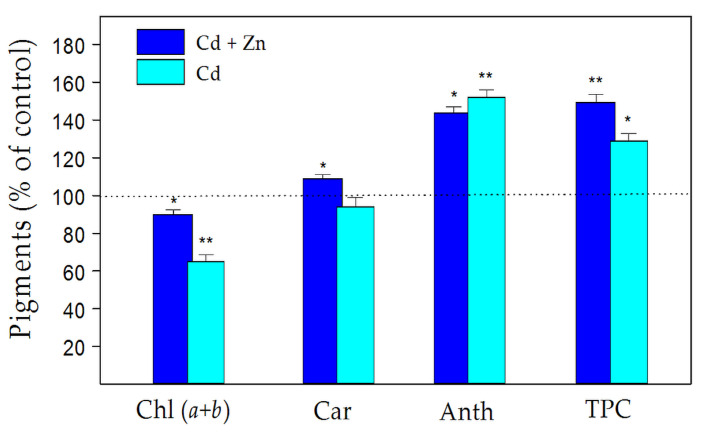
Effects of excess (900 µM) Zn on Cd-induced changes in the content of leaf pigments: the total chlorophyll content (Chl *a+b,* 100% = 2.04 ± 0.07 mg g^—1^ FW), carotenoids (Car, 100% = 0.48 ± 0.03 mg g^—1^ FW), anthocyanins (Anth, 100% = 0.31 ± 0.02 mg g^—1^ FW), and total phenolic content (TPC, 100% = 6.64 ± 0.17 mg g^—1^ FW) of *Salvia sclarea* after 8 days of exposure. Mean values (±SD, n = 3) are expressed as % from the respective controls. Asterisks indicate significant differences between control and treatments using one-way ANOVA analysis using Fisher’s LSD test (* *p* < 0.05 and ** *p* < 0.01).

Regarding the total chlorophyll (Chl *a+b*) and carotenoid (Car) contents in clary sage leaves, the Cd-only treatment caused a decrease in these pigments compared to control leaves, while the combined treatment with Zn and Cd alleviated this reduction (Figure 5). Moreover, heavy metal exposure caused a more pronounced increase in leaf anthocyanins and total phenolic content (TPC), as the Cd-only treatment had a stronger effect on the anthocyanins, while the TPC increased more after the combined treatment (Cd + Zn).

### 3.6. Changes in the Chlorophyll Fluorescence Parameters

The maximum efficiency of PSII (*Fv*/*Fm*), and the efficiency of the OEC (*Fv/Fo*) declined by 3% (*p* < 0.05) and 15%, respectively, in the Cd-only treated *S. sclarea* plants, but the excess Zn supply in the nutrient solution increased both of them to the level of control plants (Table 2). The relative PSII electron transport rate (*ETR*) also decreased under Cd treatment, while Zn supplementation in the nutrient solution resulted in a higher ETR even for the control plants.

### 3.7. P700 Photooxidation and Reduction Kinetics

The analysis of changes in the steady-state oxidation of P700 to P700^+^ and decay kinetics after turning off the FR light were used to characterize the photochemical activity of PSI. The relative changes in P700^+^ (*ΔA*/*A_830_*) for the control and treated plants are shown in Table 3. The data showed that the Cd-only treatment caused a decrease in the amount of P700^+^ (by 10,6%, *p* < 0.05), while it slightly increased (by 9%, *p* < 0.05) after the combined (Cd + Zn) exposure in comparison to the control. The dark reduction kinetics of P700^+^ were fitted by two exponential decay components, fast (with rate constant *k*_1_) and slow (with rate constants *k*_2_), as shown previously [7,47]. The results showed that the Cd-only treatment causes a significant increase (*p* < 0.01) in the fast and slow rate constants of P700^+^ dark reduction (i.e., the dark reduction times decreased) in comparison to the control plants, while the excess Zn supply alleviated these changes. Furthermore, this increase was more pronounced for the constant *k*_1_ than for *k*_2_, indicating that Cd had more influence over the fast component of the decay kinetics.

In addition, the values for microviscosity (*η*) of the thylakoid membranes were also estimated by measuring the fluorescence polarization (*P*) of the hydrophobic fluorescence probe DPH in isolated thylakoid membranes [48]. The data showed that, in comparison to the control, the relative membrane microviscosity gradually increased after the combined treatment with excess Zn and Cd, followed by the Cd-only treatment (Table 3). The results obtained herein demonstrate that the heavy metals alter the fluidity of thylakoid membranes, as Cd stress increased the microviscosity (*η*) of the thylakoid membranes (i.e., decreased the fluidity of membranes) to a greater extent than after the Zn treatment (Table 3). 

## 4. Discussion

It is assumed that the heavy metal tolerance of plant species is determined by regulating ion distribution and homeostasis, among other things [49]. The capability of trace element uptake, their efficient translocation from roots to shoots, and the pronounced ability to sequester metals are some of the characteristics that distinguish heavy metal tolerant from sensitive plants [50,51]. Our previous investigations [31] showed that the exposure of *S. sclarea* to 100 µM Cd for 8 days resulted in a high Cd accumulation in the roots and hyperaccumulation in the leaves, which were accompanied with increased Zn, Fe, Ca, and Mn uptake by the roots, and increased Fe content (by 25%) and decreased content of the other elements in the leaves. Recently published data [33] also revealed that an excess (900 µM) Zn supply in the nutrient solution (with 100 µM Cd) decreased Cd uptake and reduced Cd accumulation in both the roots and leaves. Moreover, the excess Zn treatment alone demonstrated that Zn ions can interfere with the uptake of other trace elements leading to significantly increased accumulation of Fe and Cu ions in the roots, and an increased accumulation of Fe, Mn, and Ca ions in the leaves of *S. sclarea,* which is thought to be involved in the protective strategy of clary sage plants against high Zn concentrations [32]. Similarly, other studies reported that, under excessive Zn stress, grape (*Vitis vinifera*) leaves retain high levels of Fe [52], while the content of Fe and Mg in fenugreek shoots does not change [53].

The current results demonstrate that the application of excess Zn is able to alleviate the negative effect of Cd stress on ion accumulation in *S. sclarea* plants, since co-exposure to Zn and Cd is accompanied with strongly increased translocation of Ca and Mg ions to the leaves in comparison to the Cd-only treatment and control plants (Figure 2), leading to an increased Mg content in the leaves compared to the control (Figure 1b). Moreover, in comparison to the control plants, a strongly enhanced Fe uptake by the roots and an increased Fe content in the leaves were also observed after the combined treatment (Figure 1c,d). The data obtained also showed that compared to the Cd-only treated plants under the same conditions [Table S1], co-exposure to Cd and Zn led to increased amounts of Ca (by about 17%), Mg (by 52%), Cu (by 50%), and Mn (by 53%) in the leaves of *S. sclarea* (Figure 1b,d). The increase in these essential nutrient elements in clary sage leaves after the combined treatment is most likely associated with the protective effects of an excess Zn supply to minimize Cd toxicity, as the Fe, Ca, Mg, and Mn cations have major roles in regulating (directly or indirectly) photosynthetic efficiency and oxygen-evolving activity, and are used as cofactors in many enzymes [50,54]. The observed increase in leaf Fe content after both the Cd-only and Zn-only treatments [31,32], and after the combined (Cd + Zn) treatment (Figure 1d) is most likely involved in the tolerance strategy of *S. sclarea* plants against heavy metal stress, as Fe is an essential trace element required for respiration and photosynthesis, as well as for many fundamental biological redox reactions [50,55]. Furthermore, it has been proposed that the presence of excess Fe and Mn alleviates Cd toxicity and Cd-inducible inhibition of the photosynthesis in *Oryza sativa* [56]. In plant metabolism, Mn is mostly known for its function in the OEC, where the changes in oxidation states of several Mn ions (S_0_–S_4_ redox states) in the Mn_4_Ca cluster mediate the splitting of water into protons and oxygen [44,50]. On the other hand, Ca cations are also necessary for the normal function of the OEC and for the regulation of Calvin cycle enzymes [57]. It has also been proposed that Ca plays an imperative role in controlling the membrane structure and function as it stabilizes lipid bilayers that consequently provide structural integrity to cellular membranes [58]. In addition, Cu is required for several redox reactions in plant cells, and its limitation has negative consequences on the whole metabolism, leading to decreased photosynthetic performance and diminished electron flow from PSII to PSI [50]. All of the above assumes that increased concentrations of these essential elements in plant tissues under excess Zn supply can alleviate Cd toxicity and suggests a higher tolerance of *S. sclarea* after combined exposure to Zn and Cd. 

It is generally considered that overproduction of ROS (O_2_ and H_2_O_2_) under heavy metal stress (especially Cd stress) may promote oxidative stress causing lipid peroxidation of membranes and disruption of their integrity, i.e., loss of membrane permeability and chloroplast ultrastructure [12,13,33]. Our recent study [33] showed that excess Zn application eliminated the adverse effects of Cd-induced toxicity by reducing H_2_O_2_ and MDA concentrations in leaves, leading to protection of the chloroplast ultrastructure and PSII photochemical efficiency. Similarly, the oxidative stress has been shown to decrease in *C. demersum* after Zn and Cd treatments [59] and in mung beans after zinc oxide nanoparticle (ZnO NPs) application under Cd stress [25]. The current data also revealed that co-exposure to Zn and Cd leads to lower lipid peroxidation and increased leaf cell membrane stability (MSI), and an increased antioxidant capacity compared to plants exposed to Cd alone (Table 1). As a component of antioxidant enzymes, Zn is essential for the capture of H_2_O_2_ and O_2_^•-^, and hence for reducing oxidative stress [26,60]. Furthermore, it has been demonstrated that co-exposure to excess Zn and Cd leads to a 10-fold increase in the Zn content in clary sage leaves compared to the Cd-only treatment [33]. An increase in the activities of all the antioxidant enzymes was also observed in mung bean plants after ZnO NPs application under Cd stress [25].

Medicinal plants and herbs contain free radical scavengers like phenolic compounds, which are responsible (among others) for preventing the deleterious consequences of oxidative stress, which causes lipid peroxidation and membrane damage [30,61,62,63]. Salinitro et al. [64] demonstrated that the heavy metal stress produces increased amounts of secondary metabolites in the leaves, which possess antioxidant activity. It has been suggested that polyphenols and flavonoids have antioxidant properties due to their ability to act as electron-donating agents [64,65] or to form insoluble complexes with metal cations, reducing their cellular concentrations [66]. In this regard, our data showed an increased accumulation of total phenolics (nonenzymatic antioxidants) in the leaves of *S. sclarea* induced by heavy metal application (a notion also confirmed from the presence of dark material in the leaves; Figure 3), as the TPC increases were more pronounced after the combined (Cd + Zn) treatment than the Cd-only exposure (Figure 5). Similarly, an increase in TPC was also reported for *S. sclarea* under excess Zn exposure [32] and for *Stellaria media* under Zn and Ni stress [67]. Moreover, it has been suggested [65] that plants producing high amounts of phenolic compounds under heavy metal stress could be good candidates for phytoremediation, which confirms the potential of *S. sclarea* for this purpose. 

Additionally, the anthocyanins also have high antioxidant activity, acting as scavengers of hydrogen peroxide and superoxide anions in the vacuoles [68,69]. Moreover, they can form anthocyanin–metal(n+) complexes in plant tissue to alleviate heavy metal toxicity by storing metals in peripheral cell layers [70]. Interestingly, the results showed that the contents of these protective pigments were more pronounced under Cd-only exposure than the combined treatment (Figure 5), but they cannot fully protect the leaves from Cd-induced oxidative stress and membrane injury (Table 1). This suggests that despite the increased anthocyanin content, the amount of ROS formed exceeds the protective barrier and is not sufficient to prevent Cd toxicity.

An excess Zn supply along with Cd treatment enhanced TPC in the leaves (Figure 5) probably due to an enhancement in polyphenols biosynthesis gene expression, and/or stimulation of phenylalanine ammonia lyase (PAL) enzyme activity [71]. Here, we estimated the total antioxidant capacity of clary sage determined by the FRAP and DPPH assays (Table 1). It is well established that the total phenolic compounds have significant antioxidant activities and can donate hydrogen atoms to DPPH• and scavenge it [61,72,73,74]. The stable DPPH• radical is considered to be a model of a stable lipophilic radical widely used to assess the ability of plant extracts to act as free radical scavengers or hydrogen donors, and thus to evaluate their antioxidant activity [30,72,73,74,75]. In the present study, the leaf extracts of clary sage exhibited appreciably high radical scavenging activity (DPPH) and total antioxidant capacity (FRAP), which were more pronounced after the combined treatment than the Cd-only treatment (Table 1). An increase in the antioxidant capacity by about 41% was also observed when clary sage was treated with 900 µM Zn alone. These observations imply that the higher antioxidant capacity of *S. sclarea* after the combined (Cd + Zn) exposure is most likely due to the higher phenolic contents (TPC) in *S. sclarea* leaves (Figure 5). Several investigations also demonstrated a positive correlation between the antioxidant activity and leaf phenolic content in different *Salvia* species [30,61,63,72] and other plants [62,76,77,78]. Accordingly, a previous study found that H_2_O_2_ scavenging in sage (*S. officinalis*) plants grown in heavy metal contaminated soil was more of a non-enzymatic than enzymatic process, as was observed the weak activities of the antioxidant enzymes [79].

Generally, heavy metal application affects the leaf structure and anatomy [80]. In particular, as a consequence of Cd treatment, many plants exhibited defects on their leaf structure/anatomy. For instance, pea leaves under Cd application showed cell disturbances characterized by an increase in mesophyll cell size and a reduction in intercellular spaces [5], while in willow leaves [81], the phenolic substances were accumulated in the leaf’s epidermal cells. Similarly, the current results confirm that Cd affects *S. sclarea* leaf anatomy, which exhibited a notable reduction in the intercellular spaces and an accumulation of osmiophilic granules in the vacuoles of the adaxial epidermal cells (Figure 3c). This could be linked to the increase in the amount of anthocyanins and total phenolics, as they can form complexes with metal cations, thus storing metals in the peripheral cell layers [31,70]. The excess Zn application after Cd exposure in *S. sclarea* also caused the dark material accumulation in the upper epidermis, which could be linked to the increase in the phenolic compounds. The palisade mesophyll cell structure was compromised in the plants treated with Cd only, while after the combined (Cd + Ζn) exposure, it was similar to that seen in the control plants (Figure 3). In other Cd-hyperaccumulator plants, it was also found that the leaf epidermal and mesophyll cells are sites of increased Cd accumulation/storage [82,83,84]. 

The carotenoids, which are known as one of the protectors of the photosynthetic apparatus from photodamage [53], were slightly (not statistically) increased in clary sage leaves after the combined treatment (Figure 5). The reduction in the Chl (*a+b*) content in the leaves of the Cd-only treated plants was decreased after the combined treatment (Figure 5), which corresponds with the observed increase in the leaf Mg content (Figure 1b). Similar effects on the photosynthetic pigments were found in rice genotypes under combined Zn and Cd treatments [26] and in mung beans after ZnO NPs application under Cd stress [25]. Our previous investigations showed a slightly reduced Chl *a* content in leaves of *S. sclarea* plants exposed to 900 μM Zn for 8 days [32]. Moreover, the current study shows the increase in the production of total phenols and anthocyanins combined with a concomitant reduction in the levels of photosynthetic pigments (Figure 4), which are considered biomarkers of heavy metal stress states [64,85]. All this indicates an alleviation of Cd-induced toxicity in plants after excess Zn application.

It has been suggested that the decrease in photosynthetic activity under Cd stress could be due to many factors such as a decrease in chlorophyll biosynthesis, an inhibition of the enzyme activities in the Calvin cycle, the influence of chlorophyll *a* fluorescence, and an inhibition of the photosynthetic electron transport [86]. The heavy metal induced decrease in the chlorophyll content in the leaves is proposed to be due to an inhibition of important enzymes associated with chlorophyll biosynthesis and/or an impairment of the supply of Mg and Fe ions [53]. However, unlike Cd-only treated clary sage plants (Table S1), we found that leaf Mg and Fe contents were increased after co-exposure of the plants to Zn and Cd in the nutrient solution (Figure 1b,d), suggesting that the observed slight decrease in chlorophyll content was mainly due to a decrease in the activities of enzymes related to chlorophyll biosynthesis. Since the photosynthetic pigments are important for the optimal capture of light energy in photosynthetic reactions, the chlorophyll content in the leaves is an important physiological index directly related to photosynthesis in plants. 

It is well established that the optimum value of the maximum efficiency of PSII photochemistry (*Fv*/*Fm*) is around 0.830 for vigorous plants [87,88]. In our experiment, the observed *Fv*/*Fm* values for the control and the combined (Cd + Zn) treated clary sage plants were 0.827 and 0.828, respectively, indicating that the plants were healthy and not suffering from stress (Table 2). The Cd-only treated plants showed a slight decrease in the *Fv*/*Fm* value, indicative of a mild photoinhibition. Current results from the chlorophyll *a* fluorescence analysis showed that clary sage plants co-exposed to excess Zn and Cd exhibited better PSII activity in terms of *Fv/Fm*, ETR, and the efficiency of OEC (*Fv/Fo*) in comparison to the Cd-only treated plants (Table 2). This is in accordance with our recent findings on the protective effects of excess (900 µM) Zn on Cd-induced inhibition of the PSII photochemistry (at high-light intensity), as it reduces the excess excitation energy at the PSII [33]. Moreover, excess Zn-only treatment was found to retain the photosynthetic function, and even to stimulate the activities of the PSI and PSII [32].

Furthermore, the current results show a slight decrease in the flash-induced oxygen yields after the combined treatment with excess Zn and Cd and more pronounced damping after Cd-only exposure as compared with the control (Figure 4a). The decrease in oxygen flash yields suggests a decrease in the active oxygen-evolving PSIIα centers (located in the granal domains) [7,47], which is in agreement with recent data concerning changes in the chloroplast ultrastructure and grana stacking, i.e., it was most severe after treatment with Cd alone and less so after the combined treatment with excess Zn and Cd [33]. In addition, the current results also revealed damage on the donor side of the PSII around the OEC, as the observed maximum after the 3rd flash (*Y*_3_) in the control and plants co-exposed to Zn and Cd shifted to the 4th flash after the Cd-only treatment (Figure 4a). This indicates an increase in the PSII centers in the most-reduced S_0_ state related to the lower oxidation state of the Mn cluster in the OEC [44] (Figure 4b). This finding is in accordance with the observations of Schansker et al. [89], suggesting the modification or destruction of the Mn cluster of the OEC. Similar changes in the OEC were observed in wheat plants under Cd stress [7]. Therefore, our data revealed that the Cd-only treatment leads to an increase in the blocked PSII centers, a strong decrease in the functionally active PSIIα centers in the grana domains, and to an increased amount of PSII centers in the most reduced S_0_ state, while the excess Zn supply mitigates these effects. The protection of Mn_4_Ca cluster after excess Zn application may also be due to the increased content of Mn and Ca ions in the leaves of *S. sclarea* compared to the Cd-only treated plants. In addition, no changes were detected in the OEC after treatment with 900 µM Zn alone [32]. Photo-oxidation of P700 to P700^+^ induced by FR light was used to characterize the photochemical activity of PSI [90]. It was proposed that the two components of the dark reduction kinetics of P700^+^ are due to a reduction in two different pools of PSI located in different domains of the thylakoid membranes [91]: the linear electron transport (with a constant *k*_2_) occurring in the grana domains and the cyclic electron transport around PSI (with a constant *k*_1_) occurring in the stroma lamellae. Furthermore, the rapidly operating pathway could be driven by enzymes located in the stroma lamellae, whereas enzymes mediating the slow pathway are in the grana thylakoids [90]. The rate constants of the dark reduction showed an increase in the leaves of the Cd-only treated clary sage plants in comparison to the control (Table 3), as the changes in both constants (*k*_1_ and *k*_2_) after Cd treatment with and without excess Zn are evidence for an influence on both populations of PSI (in the stroma lamellae and grana margin). Furthermore, this increase was more pronounced for *k*_1_ than for *k*_2_, indicating that Cd toxicity has more influence on the fast component of the decay kinetics (i.e., increasing the cyclic electron transport around PSI in the stroma lamellae). One reason for this could be the structural changes in the organization of thylakoid membranes, which was observed recently for the same treatments [33]. It was also demonstrated that in the Cd-only treated plants, the chloroplast ultrastructure was compromised, and the thylakoid membranes were disordered/destacked, while an excess Zn supply partially prevented these effects.

The observed strong changes in the microviscosity of thylakoid membranes after Cd-only exposure could result from changes in the lipid contents and fatty acid composition [9,10,92]. Previous studies demonstrated a decrease in the degree of fatty acid unsaturation in the chloroplast membrane caused by Cd [9,93] and a concomitant increase in the protein content [9]. It is assumed that the changes in the degree of membranes’ lipid unsaturation are either due to direct lipid peroxidation or the influence of desaturase activity [93].

## 5. Conclusions

**Figure 6:**
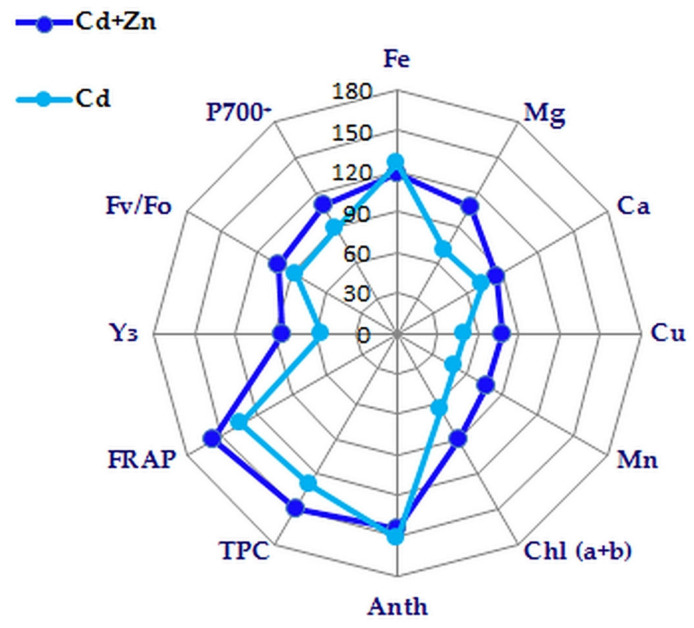
Summary of the Cd-induced changes (*p* < 0.05) with and without an excess Zn supply in some studied parameters of *Salvia sclarea* presented as % from the respective control values: the leaf content of Fe, Mg, Ca, Cu, and Mn; the leaf content of total chlorophyll (Chl *a+b*), anthocyanins (Anth), and total phenolics (TPC); the total antioxidant activity (FRAP); the maximum oxygen flash yields (*Y_3_*) and *Fv/Fo* ratio corresponding to oxygen-evolving activity; the PSI photochemical activity (P700^+^).

The current study demonstrated that the application of excess Zn along with Cd exposure in hydroponic conditions alleviated Cd toxicity in clary sage (*S. sclarea*) by enhancing: (i) the antioxidant capacity; (ii) the leaf content of total phenolics and total chlorophylls; (iii) the uptake and translocation of Fe, Ca, Mg, Cu, and Mn ions resulting in their increased leaf content, mitigating the adverse effects of Cd on the photosynthetic function and oxygen-evolving activity (Figure 6). It could be proposed that these mechanisms are involved in Zn detoxification and protection against Cd-induced structural and functional damage of the photosynthetic membranes. In summary, our results reveal that co-exposure to Zn and Cd alleviates Cd-induced alterations in the leaf anatomy, injury and fluidity of the membranes, PSI and PSII photochemical activities, and the function of the oxygen-evolution complex in clary sage plants. 

The present study highlights the potential of *S. sclarea* in both pharmaceutical applications and in the phytoremediation or phytoextraction of soils contaminated with Zn and Cd. 

## Figures and Tables

**Table 1:** Effects of excess (900 µM) Zn on the Cd-induced changes in the leaf cell membrane stability index (MSI), and the lipid peroxidation (LP) of membranes (% of control) of the clary sage (*Salvia sclarea* L.) leaves. Antioxidant capacity of the leaf extracts after exposing the plants to different treatments for 8 days measured by FRAP and DPPH assays.

Treatment (8 Days)	MSI (%)	LP (%)	FRAP (μmol Fe^2+^ g^−1^ DW)	DPPH (%)
**Control**	89.8 ± 0.5 ^a^	100 ^c^	61.87 ± 2.28 ^c^	48.38 ± 2.26 ^c^
**Cd + Zn**	74.2 ± 1.7 ^b^	124 ± 2 ^b^	97.26 ± 2.35 ^a^	67.03 ± 1.18 ^a^
**Cd**	56.9 ± 2.3 ^c^	141 ± 3 ^a^	83.15 ± 2.86 ^b^	64.35 ± 1.04 ^b^

Different letters in the same column indicate significant differences between mean values (±SD, n = 3) assessed by one-way ANOVA analysis using Fisher’s LSD post-hoc test (*p* < 0.05).

**Table 2:** Effects of the excess Zn on the Cd-induced changes in chlorophyll fluorescence parameters.

Treatment (8 Days)	Maximum Efficiency of PSII Photochemistry (*Fv*/*Fm*)	Efficiency of the Oxygen Evolving Complex (*Fv/Fo*)	Relative PSII Electron Transport Rate *(ETR)*
**Control**	0.827 ± 0.007 ^a^	4.80 ± 0.25 ^a^	57.57 ± 1.53 ^b^
**Cd + Zn**	0.828 ± 0.012 ^a^	4.83 ± 0.41 ^a^	59.11 ± 2.76 ^a^
**Cd**	0.803 ± 0.016 ^b^	4.09 ± 0.39 ^b^	55.09 ± 3.52 ^c^

Different letters between the mean values (±SD, n = 3) indicate statistically significant difference (*p* < 0.05) between treatments for the same parameter. Welch’s one-way ANOVA was performed to compare treatments on each of the chlorophyll fluorescence parameters, followed by post-hoc analysis with Games–Howell test.

**Table 3:** Effects of the excess (900 µM) Zn on the Cd-induced changes in the relative membrane microviscosity (*η*) and the PSI photo-oxidation (P700^+^) measured by relative amplitudes of FR-light induced absorbance changes (expressed as *ΔA*/*A_830_* × 10^−3^) and the rate constants of P700^+^ dark reduction (*k*_1_ and *k*_2_) in leaves of *Salvia sclarea*.

Treatment (8 Days)	P700^+^ (*ΔA*/*A_830_* × 10^−3^)	*k*_1_ (s^−1^)	*k*_2_ (s^−1^)	*η*
**Control**	11.71 ± 0.38 ^b^	0.55 ± 0.07 ^c^	0.12 ± 0.02 ^c^	2.946 ± 0.11 ^c^
**Cd + Zn**	12.78 ± 0.44 ^a^	0.87 ± 0.09 ^b^	0.16 ± 0.01 ^b^	3.257 ± 0.15 ^b^
**Cd**	10.47 ± 0.27 ^c^	1.29 ± 0.05 ^a^	0.21 ± 0.01 ^a^	3.680 ± 0.17 ^a^

Different letters in the same column indicate statistically significant differences (*p* < 0.05) between mean values (±SD, n = 3) assessed by one-way ANOVA analysis using Fisher’s LSD test.

## Data Availability

The data presented in this study are available in this article.

## Supplementary Materials

The following supporting information can be downloaded at: https://www.mdpi.com/article/10.3390/plants11182407/s1, Table S1: Content of the trace elements in the *Salvia sclarea* tissues determined after 8 days of 100 µM Cd-only exposure.
