# Supplementary material for: Combined Impact of Excess Zinc and Cadmium on Elemental Uptake, Leaf Anatomy and Pigments, Antioxidant Capacity, and Function of Photosynthetic Apparatus in Clary Sage (Salvia sclarea L.)
Source: Plants (Basel). 2022 Sep 15;11(18):2407. doi: 10.3390/plants11182407 (PMC9500708; doi:10.3390/plants11182407)
Supplement: Supplementary file 1 [file plants-11-02407-s001.zip › plants-1819179-supplementary.pdf]

Combined Impact of Excess Zinc and Cadmium on Elemental Uptake, Leaf Anatomy and Pigments, Antioxidant Capacity, and Function of Photosynthetic Apparatus in Clary Sage (*Salvia sclarea* L.)

Anelia Dobrikova, Emilia Apostolova, Ioannis-Dimosthenis S. Adamakis, Anetta Hanć, Ilektra Sperdouli and Michael Moustakas

Table S1. Content of the trace elements in the *Salvia sclarea* tissues (μg g^{-1} DW) determined after 8 days of 100 μM Cd exposure [#]. Values are means with standard deviation (± SD). Asterisks (*$p < 0.05$; ** $p < 0.01$) indicate statistical differences between corresponding values for the control and Cd-treated samples.

	**Fe**	**Mn**	**Cu**	**Mg**	**Ca**
Leaves					
Control	77,2 ±2,3	37,2 ±1,1	11,6 ±0,3	4377 ±131	8531 ±252
+ Cd	96,5 ±2,9*	18,1 ±0,5*	5,8 ±0,2*	3079 ±102*	6175 ±181*
Roots					
Control	561 ±17	40,8 ±1,2	20,8 ±0,6	3273 ±107	6214 ±186
+ Cd	1234 ±36**	47,7 ±1,4*	228,5 ±6,7**	1759 ±53**	8484 ±254*

[#] The data are from Reference [31].
